# Efficacy of adalimumab in young children with juvenile idiopathic arthritis and chronic uveitis: a case series

**DOI:** 10.1186/1756-0500-7-316

**Published:** 2014-05-24

**Authors:** Francesco La Torre, Marco Cattalini, Barbara Teruzzi, Antonella Meini, Fulvio Moramarco, Florenzo Iannone

**Affiliations:** 1 Department of Paediatrics, Antonio Perrino Hospital, Brindisi, Italy; 2 Pediatric Clinic University of Brescia and Spedali Civili di Brescia, Brescia, Italy; 3 Rheumatology Unit, L. Sacco University Hospital, Milan, Italy; 4 Policlinic Hospital, Rheumatology Unit, University of Bari, Bari, Italy

**Keywords:** Adalimumab, Juvenile idiopathic arthritis, Children, Uveitis

## Abstract

**Background:**

Juvenile idiopathic arthritis is a relatively common chronic disease of childhood, and is associated with persistent morbidity and extra-articular complications, one of the most common being uveitis. The introduction of biologic therapies, particularly those blocking the inflammatory mediator tumor necrosis factor-α, provided a new treatment option for juvenile idiopathic arthritis patients who were refractory to standard therapy such as non-steroidal anti-inflammatory drugs, corticosteroids and/or methotrexate.

**Case presentations:**

The first case was a 2-year-old girl with juvenile idiopathic arthritis and uveitis who failed to respond to treatment with anti-inflammatories, low-dose corticosteroids and methotrexate, and had growth retardation. Adalimumab 24 mg/m^2^ every 2 weeks and prednisone 0.5 mg/kg/day were added to methotrexate therapy; steroid tapering and withdrawal started after 1 month. After 2 months the patient showed good control of articular and ocular manifestations, and she remained in remission for 1 year, receiving adalimumab and methotrexate with no side effects, and showing significant improvement in growth. Case 2 was a 9-year-old boy with an 8-year history of juvenile idiopathic arthritis and uveitis that initially responded to infliximab, but relapse occurred after 2 years off therapy. After switching to adalimumab, and adjusting doses of both adalimumab and methotrexate based on body surface area, the patient showed good response and corticosteroids were tapered and withdrawn after 6 months; the patient remained in remission taking adalimumab and methotrexate. The final case was a 5-year-old girl with juvenile idiopathic arthritis for whom adalimumab was added to methotrexate therapy after three flares of uveitis. The patient had two subsequent episodes of uveitis that responded well to local therapy, but was then free of both juvenile idiopathic arthritis and uveitis symptoms, allowing methotrexate and then adalimumab to be stopped; the patient remained in drug-free remission.

**Conclusion:**

This report includes the first published case of the use of adalimumab in a child aged <3 years. Our clinical experience further supports the use of biologic therapy for the management of juvenile idiopathic arthritis and uveitis in children as young as two years of age.

## Background

Juvenile idiopathic arthritis (JIA) is defined as arthritis of unknown etiology with onset prior to 16 years of age that persists for 6 weeks or longer and is not secondary to any other conditions [1]. Classification of JIA is based on the criteria developed by the International League of Associations for Rheumatology (ILAR) [1,2]. JIA is one of the more common chronic diseases of childhood [3], and its prevalence in the United States (US) and Northern Europe is estimated to be 7–21 cases per 100,000 of population [4]. JIA can have an adverse effect on the growth and development of joints and bones, often persists into adulthood and can result in significant long-term morbidity, including physical disability [3,5,6]. Factors contributing to growth suppression in JIA include the degree, extent and duration of disease activity, age at onset, immobility, suboptimal nutrition and systemic corticosteroid therapy [5].

Standard medical treatment for JIA consists of non-steroidal anti-inflammatory drugs (NSAIDs), systemic glucocorticoids and/or disease-modifying anti-rheumatic drugs (DMARDs) such as methotrexate. However, up to 30% of patients do not respond to treatment [7]. A new era in the treatment of JIA arrived with the introduction of biologic therapies, particularly those blocking the inflammatory mediator tumor necrosis factor-α (TNF-α) [8]. Approximately 80% of patients with active polyarticular disease, despite previous use of non-biologic DMARDs, have been shown to respond to biological drugs, and children treated with biologic therapy feel better and have less pain [9,10]. Treatment guidelines in the US now recommend switching to biologic therapy in JIA patients with persistent moderate-to-severe disease activity, or drug intolerance, after four months of treatment with standard medical therapy [11]. TNF-α has also been reported to play a role in the pathogenesis of ocular inflammation, with high levels detected in the serum and aqueous humor of patients with uveitis [12-14]. Chronic anterior uveitis is insidious, highly refractory, bilateral in 80% of patients, often corticosteroid-dependent, and associated with high rates of medium- and long-term complications such as posterior synechiae, band keratopathy, cataract and glaucoma [15,16]. Overall, 20% to 25% of all pediatric uveitis is associated with JIA [17]. Anterior uveitis is the most frequent extra-articular disease associated with JIA [15,18,19]. The major risk factors for the development of uveitis in JIA patients are oligoarticular pattern of arthritis, onset of arthritis before 7 years of age and antinuclear antibody positivity [18]. In the initial stages of mild to moderate inflammation, uveitis is entirely asymptomatic. This has led to the current practice of screening all children with JIA regularly for uveitis. Approximately 12% to 38% of patients with JIA will develop uveitis in the 7 years following the onset of arthritis [19]. In 30% to 50% of children with JIA-associated uveitis, structural complications are present at the time of diagnosis [20]. Furthermore, about 50% to 75% of those with severe uveitis will eventually develop visual impairment secondary to ocular complications such as cataract, glaucoma, band keratopathy and macular pathology [21-23]. Early and aggressive intervention is appropriate to prevent irreversible complications and preserve visual acuity.

Significant prognosticators of poor visual acuity include structural changes at presentation, the need for intraocular surgery, posterior segment inflammation, abnormal intraocular pressure and failure to maintain long-term disease control as marked by persistent anterior chamber (AC) cell scores of 1 or higher [20-22,24].

While anti-TNF agents have a significant impact on the management of JIA, experience on their effects on uveitis remains limited, particularly in younger patients. The three cases presented here document the safety and effectiveness of adalimumab in children with JIA and co-morbid uveitis, one of them being, to our knowledge, the first published case report of the use of this biological drug in a child aged <3 years.

### Case Presentations

#### Case 1

**Figure 1:**
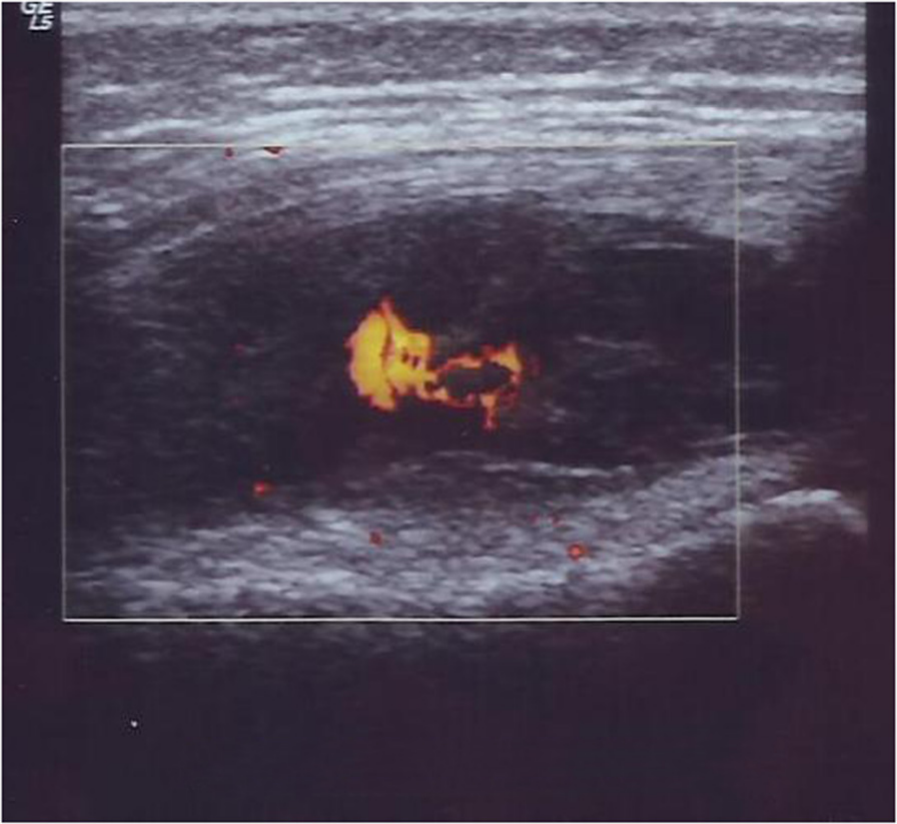
**Case 1 left knee ultrasound.** Transverse gray-scale and color Doppler 12–5 MHz ultrasound image obtained over the medial aspect of the knee showing abundant synovial pannus filling the subquadriceps recess as a band of hypoechoic tissue intermingled with fluid. Marked synovial hyperemia is observed at color Doppler examination indicating active pannus.

**Figure 2:**
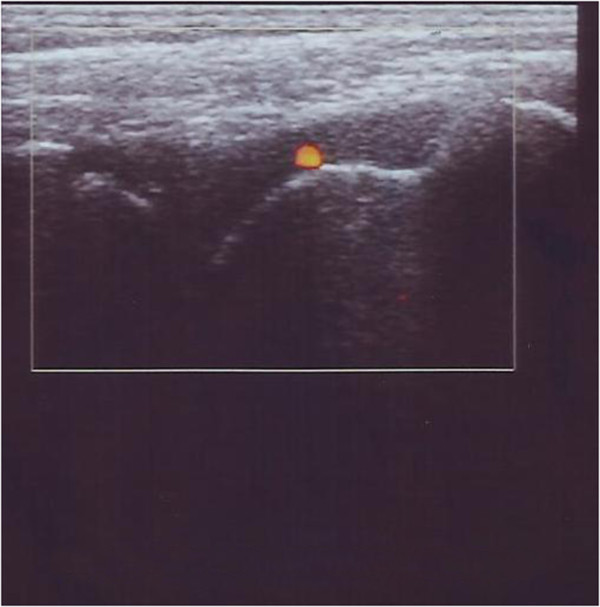
**Case 1 left ankle ultrasound.** Midsagittal 12–5 MHz ultrasound image over the dorsal ankle shows a distended anterior joint recess, filled with hypoechoic fluid with active synovial pannus as marked by color Doppler examination.

**Figure 3:**
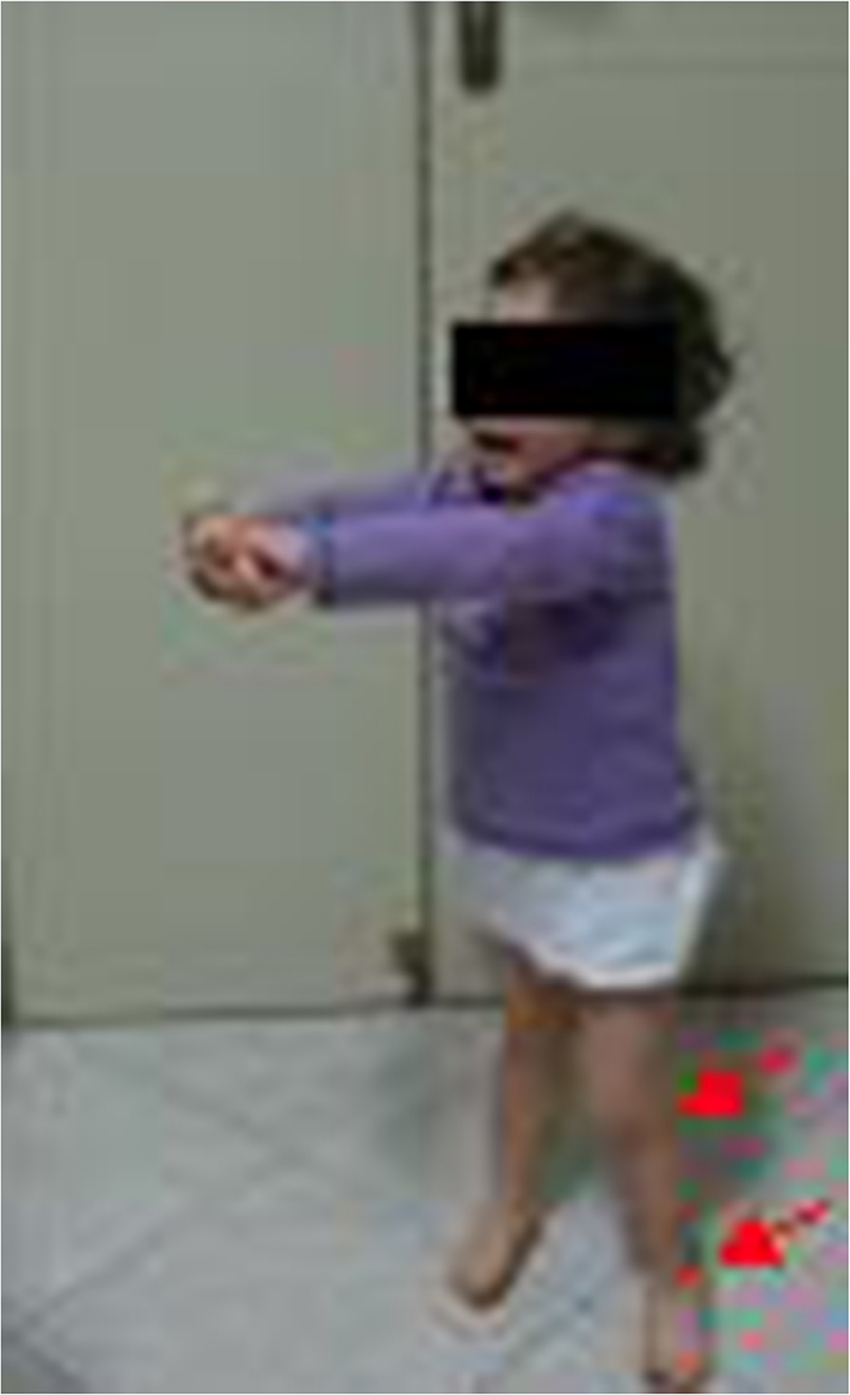
**Case 1 (age 2 years).** Photograph patient with juvenile idiopathic arthritis with fixed flexion contraction of the left leg for active knee and ankle joints arthritis (red arrows).

A 2-year-old girl presented at hospital with JIA and bilateral chronic uveitis. The onset of articular disease was at 8 months of age and the diagnosis of JIA was made at age 14 months. She had previously been treated with injections of triamcinolone hexacetonide into her left knee and left ankle joints, which resulted in temporary disease control, but arthritis relapsed in the same joints after 3 months (Figures 1 and 2). The patient had difficulties in walking and climbing stairs. Anti-inflammatory drugs and low-dose corticosteroids were initiated without benefit. Two months later, at age 19 months, subcutaneous methotrexate 15 mg/m^2^/week was added. Arthritis improved, but bilateral chronic anterior uveitis developed after 3 months, despite ongoing methotrexate treatment. The diagnosis was made at the regular ocular screening follow-up, because she didn’t show any ocular symptoms, but at slit-lamp examination cells in the anterior chamber were present. Uveitis was initially managed with standard eye care, but several relapses occurred resulting in the formation of bilateral papilledema. Brain magnetic resonance imaging was normal. She had growth retardation (weight 9.8 kg[<3^rd^ percentile], height 76 cm[−3 standard deviations from normal]) (Figure 3).

**Figure 4:**
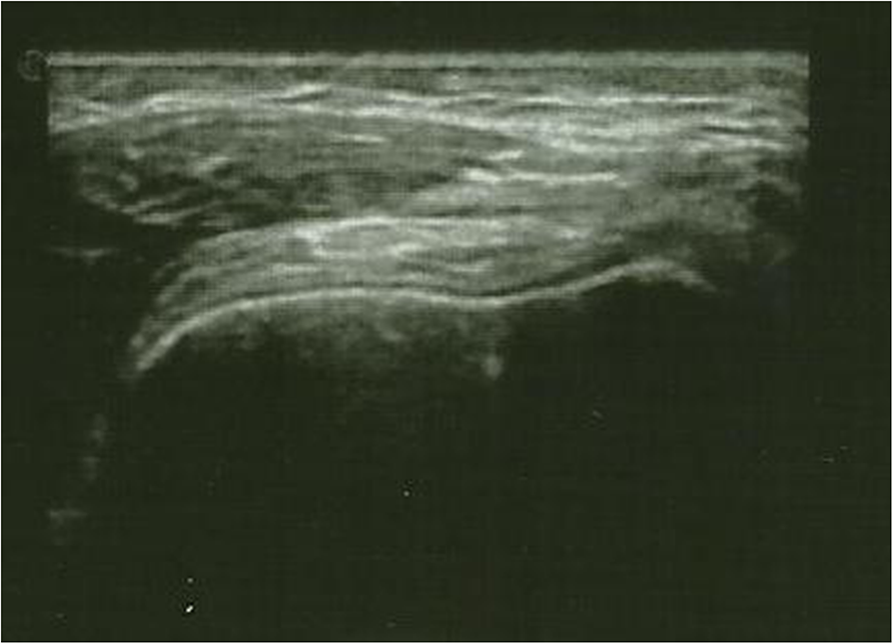
**Case 1 left knee ultrasound after treatment.** Left knee: transverse gray-scale 12–5 MHz ultrasound image obtained over the medial aspect of the knee showing normal aspect of the joint without fluid or synovial pannus into the subquadriceps recess.

**Figure 5:**
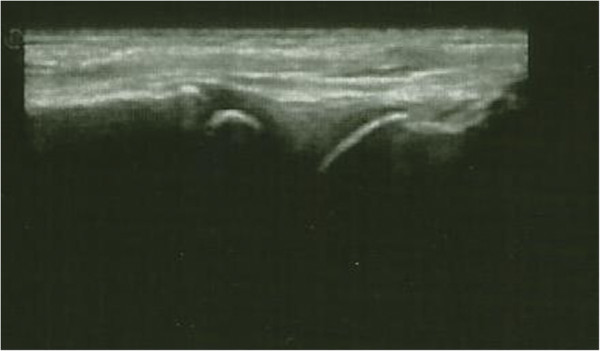
**Case 1 left ankle ultrasound after treatment.** Midsagittal12 − 5 MHz US image over the dorsal ankle showing normal joint recess, without hypoechoic fluid or synovial pannus.

**Figure 6:**
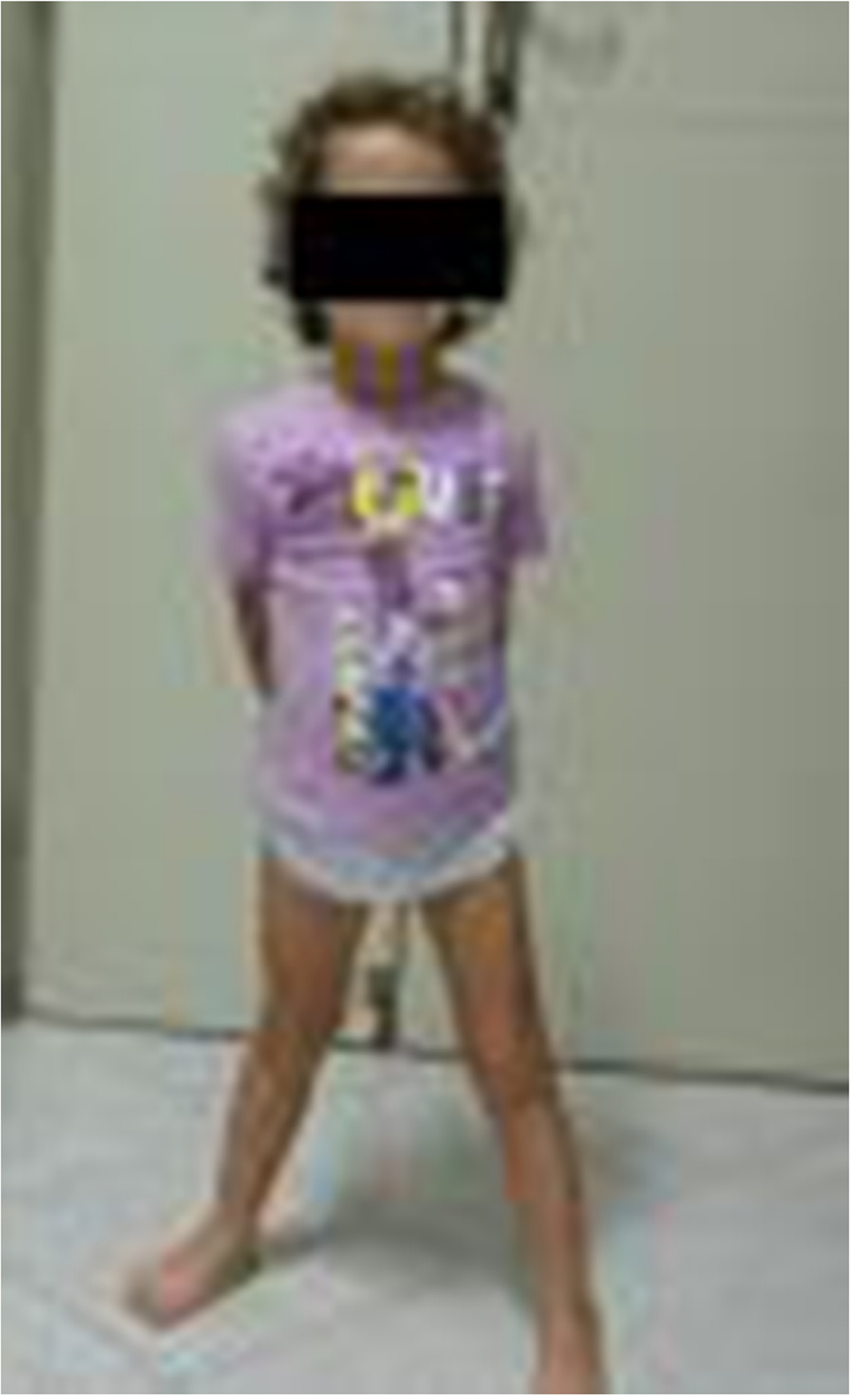
**Case 1 after 2 years of ongoing adalimumab treatment.** Photograph showing patient in remission at 4 years of age.

The patient was started on treatment with adalimumab 24 mg/m^2^ every 2 weeks in combination with prednisone 0.5 mg/kg/day for one month with subsequent steroid tapering; methotrexate was continued at the same dose. Two months later, both articular and ocular manifestations were well controlled, papilledema had resolved and corticosteroid treatment was stopped (Figures 4 and 5). One year later the patient was still in remission receiving adalimumab and methotrexate, without any side effects. Her growth rate significantly improved in the absence of corticosteroid treatment and with no disease relapses (weight 15 kg[10^th^ percentile], height 100 cm[−1 standard deviation from normal]) (Figure 6). After two years, methotrexate dose reduction was undertaken.

### Case 2

A boy aged 9 years and 4 months was admitted to hospital in June 2012 with oligo-articular extended-onset JIA and associated chronic bilateral uveitis with posterior synechiae. Immune serology was positive for antinuclear antibodies (ANA) and human lymphocyte antigen (HLA) B27, while rheumatoid factor (RF) was negative. Age at disease onset was 1 year 9 months, when the patient presented with bilateral anterior uveitis with eyes redness and headache, diagnosed with slit-lamp that showed cells in the anterior chamber. He was treated with steroid eye drops and mydriatics. At the same time, he presented arthritis of the left ankle, progressing into oligoarticular extended form with arthritis of the knees, left elbow, left shoulder, left wrist and the small joints of the hands and feet. The patient was treated with injections of triamcinolone hexacetonide1 mg/kg into the left ankle and both knees.

At 2 years of age (July 2005) he had a bilateral uveitis flare and was started on oral prednisone 1 mg/kg/day and subcutaneous methotrexate 10 mg/m^2^/week. Between November 2008 and July 2011 the patient was treated with infliximab, and had a good response for 2 years, but then experienced a number of relapses of uveitis. Therefore, in August 2011, he was switched to adalimumab 20 mg every 2 weeks.

On his most recent presentation, the patient was admitted to hospital with relapsed uveitis and arthritis of the right knee (limited range of motion and pain) while being treated with prednisone 0.5 mg/kg/day, adalimumab 20 mg every 2 weeks and methotrexate 10 mg/week. In October 2012, the dosages of both adalimumab and methotrexate were progressively increased based on body surface area (m^2^): adalimumb to 25 mg every 2 weeks (specific paediatric formulation 24 mg/m^2^ every 2 weeks) and methotrexate to 15 mg/week(15 mg/m^2^/week). The corticosteroid dosage was progressively tapered to withdrawal over the next 6 months without any flare of uveitis. The patient has maintained clinical remission for the last 6 months receiving ongoing therapy with adalimumab 24 mg/m^2^ every 2 weeks, based on surface area using specific pediatric formulation, and with MTX 15 mg/m^2^/week.

### Case 3

A 5-year-old girl was referred in August 2008 for management of arthritis. The patient had severe swelling and limited range of motion in the right ankle, slight swelling and slight effusion in the right knee with full range of motion, and slight swelling of the right wrist and the fourth metacarpo-phalangeal joint of the left hand. Physical examination was otherwise unremarkable. Laboratory tests showed high ESR (64 mm/h) and ANA positivity, with a titer of 1:640 and homogeneous pattern. Though in the absence of ocular symptoms, eye evaluation with slit-lamp examination was positive for signs of iridociclitis, showing cells in the anterior chamber with initial band keratopathy and sinechiae; visual acuity was normal. A diagnosis of ANA positive-JIA was made and naproxen, corticosteroid eye drops and mydriatics were prescribed. Three weeks later the patient showed a partial response to treatment, and slit-lamp examination showed no signs of active uveitis but persistent band keratopathy and sinechie. Subcutaneous methotrexate 12.5 mg/m^2^/week was then initiated.

In October 2008 physical exam revealed severe swelling of the right wrist with limitation in the range of motion, and swelling of the fourth left metacarpo-phalangeal joint. The diagnosis of polyarticular JIA was confirmed, NSAIDS were stopped and prednisone 1 mg/kg/day was started then rapidly tapered over the next two weeks. Two months later the patient showed a good response with an unremarkable physical examination and no active uveitis. From May 2010 to September 2010 the patient experienced three flares of uveitis that were treated with local corticosteroids and mydriatics; physical exam on these occasions revealed no signs of arthritis and laboratory findings were normal. However, given the high rate of uveitis flares, the decision was made to add subcutaneous adalimumab 24 mg/m^2^ every 2 weeks to the ongoing therapy with methotrexate. After two other episodes of uveitis (November 2010 and March 2011) that responded well to local therapy, the patient has shown no signs of either arthritis or uveitis during regular follow-up since March 2011. Methotrexate therapy was tapered and then stopped in December 2012, and adalimumab was continued. In February 2013 adalimumab was stopped and the patient has remained free of arthritis and uveitis since then. Both methotrexate and adalimumab were well tolerated, with no clinical or laboratory signs of adverse effects.

## Discussion

We have described three children with JIA and uveitis who responded well to adalimumab therapy. All were treated with the pediatric formulation of adalimumab with dosing based on body surface area. The main evidence for use of adalimumab in children with JIA has come from a randomized, controlled trial in patients with polyarticular disease [25]. Other anti-TNF agents have also shown benefits in these patients [26,27].

A critical issue in the management of JIA is co-morbid uveitis that is often refractory to conventional DMARDs and require high dose glucocorticoids. Recently, anti-TNF agents, including infliximab and adalimumab, have shown to be very effective in JIA patients who present with refractory uveitis [28-38].

Although an indirect comparison showed that adalimumab and infliximab were equally efficacious in terms of response to treatment and for the prevention of disease flare, adalimumab appeared to be more effective and better tolerated than infliximab, as evidenced by recent data from the National Italian Registry of childhood chronic uveitis associated with JIA and from other published experiences [38-40]. Moreover, an open-label prospective, comparative, multicenter cohort study of childhood noninfectious chronic uveitis, with 22 children with uveitis associated with JIA (12 treated with Adalimumab and 10 with Infliximab), confirmed that over 3 years of treatment, adalimumab is more efficacious than infliximab in maintaining remission of chronic childhood uveitis [36]. Recently, Simonini *et al.*, even if limited to a relatively small group, showed a better efficacy of Adalimumab when used as first anti- TNF alpha treatment in childhood chronic uveitis [37].

In contrast, etanercept has been reported not to halt the onset of uveitis or to be more effective than placebo [41,42], and some reports of new-onset uveitis associated with etanercept use in JIA have been published [43].

In fact, infliximab has been shown to be better than etanercept in children with ankylosing spondylitis and anterior uveitis and, in patients with JIA and uveitis, adalimumab showed better outcome than etanercept [28,44]. For these reasons, etanercept is not considered to be effective in treating intraocular inflammation [45].

We hypothesize that these monoclonal antibodies (adalimumab and infliximab) would be more effective in suppressing ocular inflammation than etanercept, which is a synthetic receptor for TNF-α and does not act against cell-bound TNF.

We also suppose that adalimumab would provide a superior response than infliximab in maintaining remission, because it binds to TNF-α on the cell surface and not just in the circulation.

For the reasons mentioned above, adalimumab was chosen in these three patients. Patient 2 in this case series had a better response to adalimumab therapy after loss of efficacy of infliximab. Increasing the dosage of adalimumab based on body surface area resulted in achievement of good therapeutic activity in this patient. When treating JIA, it is recommended that methotrexate is given with adalimumab to optimize outcomes [25]. All three cases in this report were receiving methotrexate at the time of adalimumab initiation.

Both methotrexate and adalimumab have corticosteroid-sparing properties, and systemic corticosteroid therapy was tapered and then withdrawn in all three cases. This was particularly notable in case 2, who had corticosteroid-dependent disease. Withdrawal of corticosteroids in the youngest patient (case 1) allowed catch-up growth to occur. The positive effect of anti-TNF agents on disease control, and subsequently on the growth of children with JIA, is a particularly attractive feature of this approach to JIA management. Besides, in case 1 patient, when treatment was initiated, use of adalimumab in the 2-year-old girl was off-label, because, at that time, adalimumab was only approved in Italy for use in JIA patients aged 4–17 years. But now, the authorization is extended for JIA patients over 2 years old.

Certainly, good results in terms of uveitis outcome were seen in our three cases. A recent survey of US pediatric rheumatologists on the treatment of patients with JIA revealed that anti-TNF agents were used more often in those with versus without co-morbid uveitis [40]. The co-existence of JIA and uveitis was a contributing factor to the decision to use adalimumab in case 3. This patient was then able to stop methotrexate treatment, and ultimately adalimumab, without relapse of either JIA or uveitis. However, the follow-up period is not yet long enough to make a definite statement about the ability of adalimumab to induce long-term drug-free remission in this patient. The other two patients achieved remission and continued to receive adalimumab and methotrexate.

Our three patients tolerated adalimumab very well; no clinical or laboratory adverse events were documented. There are, however, well-known risks associated with anti-TNF agents, including re-activation of latent tuberculosis infection. This risk is managed by pre-treatment screening. In addition, malignancy has been reported in children who have been treated with adalimumab, etanercept or infliximab [46]. However it is difficult to determine if this risk is due to the biologic therapy or determined by the concomitant use of other immunosuppressive drugs, or by the higher risk of malignancy in patients with JIA [47]. Such data underscore the importance of large-scale, coordinated, national and international pharmaco-vigilance programs for better quantifying the risks and benefits of anti-TNF therapy in children. Future analyses also need to take into account the high cost of biological therapies, and therefore the cost-effectiveness of this approach to treatment.

## Conclusions

We have reported herein the case details of three children with JIA and chronic uveitis treated with adalimumab. These three children had good outcomes after the addition of adalimumab to their treatment regimen. Our data, from routine clinical practice, and in agreement with clinical trials, further support the use of biologic therapy for the management of JIA and uveitis, not responder to DMARDs, in children over two years of age. Long-term follow-up, both from registries and clinical trials, will help to further elucidate the ongoing efficacy and tolerability of adalimumab, and other biologic drugs, in these patients.

## Consent

Written informed consent was obtained from the patients’ parents or legal guardians for publication of these Case Reports and any accompanying images. A copy of the written consent is available for review by the Editor-in-Chief of this journal.

## Abbreviations

ANA: Antinuclear antibody; DMARDs: Disease-modifying anti-rheumatic drugs; ESR: Erythrocyte sedimentation rate; HLA: Human lymphocyte antigen; ILAR: International League Against Rheumatism; JIA: Juvenile idiopathic arthritis; NSAIDs: Non-steroidal anti-inflammatory drugs; RF: Rheumatoid factor; TNF-α: Tumour necrosis factor-alpha.

## Competing interests

The authors declare that they have no competing interests.

## Authors’ contributions

All the authors were involved in this report. FLT and FI wrote the manuscript, FLT and FM cared for the case 1 patient. BT cared for case 2 patient, BT was responsible for data collection and manuscript preparation.MC and AM were involved in the care of patient 3, MC was responsible for data collection and manuscript preparation. All authors read and approved the final manuscript.
